# Adrenalin and It’s Uses in General Surgery

**Published:** 1903-12

**Authors:** 


### Adrenalin and It's Uses in General Surgery.

---

Under the above title an article appears in the October issue of the *Indian Medical Gazette,* from the pen of Harry Gidney, F. R C. S. (Edin.), D. P. H. (Camb.), etc. The author finds that "the clinical usefulness of Adrenalin is very great and extensive, and owing to its power of rapidity and effectively producing vaso-motor constriction, it is adapted to the treatment of all inflammatory conditions. The drug is also of extreme value in arresting hemorrhage during all surgical operations. It is indicated whenever and wherever any local hyperæmia exists, more especially in inflammations of mucous surfaces, such as those of the eye, throat, larynx, pharynx, urethra, bladder, nose, rectum, vagina, uterus, stomach, etc. It is used not only to stay hemorrhage when it exists, but also as a preventive or controlling remedy, given either internally or externally prior to an operation, so as to lessen the amount of bleeding during the performance of that operation. It is a non-irritant to mucous membrane unless when used too frequently and in excess.

"On reading the literature on the subject," says the writer, "I find that Adrenalin is admitted to be the most powerful and rapid cardiac stimulant and toxic we have, being chiefly used in cardiac affections, hæmatemesis, hemoptysis, hemophilia, hematuria, menorrhagia, post-partum hemorrhage, purpura, scurvy, etc. It is said to be the most rapid restorative in chloroform and other forms of anesthetic syncope, and in such cases it is advisable to administer it intravenously."

The author resports the results of several operations, major and minor, in which Adrenalin was employed. The first case was one of fracture of the vertex of the skull. As one of the larger branches of the middle meningeal artery had been torn there was profuse dural hemorrhage and capillary oozing, which were controlled by the use of the 1-1000 solution. In the second case, one of hemorrhoids, profuse bleeding was checked by the rectal insertion of a plug of cotton wool soaked with Adrenalin Chloride Solution.

The third case was one of skin grafting in which the author tried pressure to stop the capillary bleeding. As the procedure was somewhat tedious he applied Adrenalin Chloride Solution with almost immediate cessation of all oozing, and what is usually a lengthy and sanguinary operation was converted into a short and comparatively bloodless one.

The fourth case, one of hemorrhage after the extraction of teeth, and the fifth, which appears to embrace the author's experience in a number of cases of opistaxis, afforded additional opportunity to test the hemostatic effect of Adrenalin.

In case VI a post-partum hemorrhage was checked by swabbing the uterine cavity with Adrenalin Solution, while the same happy result was obtained in a case of secondary hemorrhage following an operation for the relief of a mammary abscess.

The author has found that the instillation of a 1-5000 to 1-2000 solution of this drug reduces the inflammation and considerably cuts short the process of conjunctivitis. He usually applies it (diluted) over the inflamed parts by means of a soft camel's-hair brush. He always uses the preparation containing Chloretone, which has a decided local anesthetic action relieving much of the photophobia and pain. He is fully convinced of the power of Adrenalin to arrest or lessen the bleeding that arises from the cut ends of the iris after iridectomy. He speaks highly of its efficiency in chemosis, cataract operations, evisceration of the eyeball, operations for ectropion, symblepharon and trachomatous pannus.

The author concludes that in all cases of minor surgery in which it is desired to arrest bleeding from any cut or exposed surface we have in Adrenalin a most useful, powerful and rapid drug—one that is non-poisonous, non-irritant and non-accumulative, especially in operations upon the conjunctiva and eyelids.

